# Reference values and associated factors of hand grip strength in elderly Saudi population: a cross-sectional study

**DOI:** 10.1186/s12877-019-1288-7

**Published:** 2019-10-16

**Authors:** Bader Alqahtani, Aqeel Alenazi, Mohammed Alshehri, Mohammed Alqahtani, Ragab Elnaggar

**Affiliations:** 1 Department of Rehabilitation Sciences, College of Applied Medical Sciences, Prince Sattam Bin Abdulaziz University, Al-Kharj, 11942 Kingdom of Saudi Arabia; 2 Department of Rehabilitation Sciences, College of Applied Medical Sciences, Jazan University, Jazan, Kingdom of Saudi Arabia; 3 College of Medicine, Dar Al Uloom University, Riyadh, Saudi Arabia; 4 Department of Physical Therapy, Cairo University, Cairo, Egypt

**Keywords:** Handgrip strength, Saudi, Older adults

## Abstract

**Background:**

Hand grip strength (HGS) is an important function of upper extremities for older adults. Several studies have shown the importance of measuring HGS in different settings. Current established normative values of HGS are applicable for Western countries. However, there is limited information of normative values of HGS after considering demographics in Saudi population. Therefore, this study aimed to establish normative values of HGS stratified by age and gender, and to determine the association of anthropometric measurements with the HGS in Saudi population.

**Methods:**

A cross-sectional study included a total of 1048 participants (mean age 73 ± 5 years). Grip strength was calculated by the average peak force of three trials for the dominant hand using a dynamometer. Sociodemographic data on age, gender, marital status, educational levels were collected. Anthropometric measurements including height, body mass index, arm circumference, and upper arm length were obtained. The sample was categorized into three age groups: 65–69 years, 70–74 years, and 75–80 years. Linear regression analysis was used to assess the association between the sociodemographic and anthropometric data and HGS.

**Results:**

The mean values of HGS (kg) for men for each age group were 36.9 ± 8.3 for the younger group, 35.7 ± 7.4 for the 70–74 years group and 30.5 ± 7.1 for the older group. The mean values of HGS for women for each group were 23.2 ± 4.7 for the younger group, 21.1 ± 4.6 for the 70–74 years group and 18.8 ± 4.9 for the older group. The HGS was negatively associated with the age for men (B = -.40, 95% confidence interval (CI) [−.52, −.29], *p* < 0.001) and women (B = -.30, 95% CI [− 0.38, − 0.22], p < 0.001), and positively associated with the arm length in men (B = .87, 95% CI [.60, 1.15], p < 0.001). The HGS was positively associated with the educational level in men (B = .66, 95% CI [.09,1.21], *p* = .02), but negatively associated in women (B = -.42, 95% CI [− 0.75, − 0.08], *p* = .01).

**Conclusion:**

This study is the first that established normative values of HGS for older adults in Saudi Arabia. Future research may benefit from the current normative value of HGS in Saudi population for geriatric rehabilitation programs.

## Background

The hands are the most functional part of the upper extremities and also referred to as the most sophisticated and differentiated musculoskeletal tool in the human [1]. Measuring hand grip strength (HGS) is important to understand upper limb function and work capacity. Additionally, for people with paired HGS due various systemic pathologies, recognizing the effectiveness of several therapeutic modalities on HGS should be considered [2, 3]. Various methods have emerged to objectively evaluate the factors related to individuals’ ability to use their hands effectively in daily and work activities. However, the HGS assessment is one of these useful evaluations [3].

HGS is a feasible tool to evaluate prognosis of different conditions in clinical settings. HGS has been shown to be a prognostic factor in the general population and in people with chronic diseases [4]. Previous evidence has shown that HGS is an independent predictor for all-cause mortality, independent of known confounding factors such as socioeconomic factors and physical activity [5]. In addition, weak HGS has shown to be associated with high fatality rates in individuals with major illnesses [6–8]. HGS is associated with muscle strength of other muscle groups including lower extremities [9]. The simplicity, portability, and low cost make HGS a useful tool for clinical and epidemiological research to avoid several complications related to health.

Numerous studies have been published about the normative values for HGS in the general population with different age groups [3, 10–14]. However, few studies have reported the normative data for HGS in older adults. Hands usually undergo some anatomical and physiological changes with aging. Recent research have shown normative values for HGS in older adults in different countries including Brazil [15], China [16] and other western countries [17–19]. However, suggested normative values of these studies might not be applicable in Middle East countries such as Saudi Arabia because of differences in sociocultural aspects [20]. Previous research has concluded that HGS differs across different regions in older adults [6]. These differences might be attributed to the variations in skeletal muscle mass according to different ethnicities, sociocultural factors, and leisure activities. Therefore, it is important to establish normative values for HGS for older adults by regions such as Saudi Arabia.

To our knowledge, there is a limited research about normative data and associated factors that affect HGS in Saudi older adults. Only one study has included data for HGS from the Saudi population, and only for people aged 61 to 70 as subgroup [6]. Older adult age groups can range from 65 to 80 years old; thus, normative data should include high range of age groups. In addition, there is lack of research about factors that may influence HGS in the middle east such as age, gender, mass of arm muscles, as these factors have shown association with HGS in different countries [10, 12, 21, 22]. While the comparison to normal data of HGS is important to make clinical decisions about treatment choices, attainment of normative data for the HGS in elderly was the main topic in many studies. Far as we know, most of the normative data are based on western literature which may not be applicable to the Saudi population. Thus, the primary purpose of the present study was to establish normative values stratified by age and gender, and to determine the association of demographics and anthropometric measurements such as weight, height, and body mass index (BMI), arm circumference, and upper arm length with the HGS in Saudi population.

## Methods

### Design and study participants

A community-based cross-sectional study was carried out in the Riyadh region, Saudi Arabia, included a total of 1048 participants (mean age 73 ± 5 years), between September 2017 and December 2018. All subjects were recruited using a convenience sample of older adults living in the community. A minimum sample size of 926 participants was needed to achieve 90% power and to detect an effect size (Cohen’s f^2^) of 0.02 attributable to 7 independent variables using an F-Test (multiple regression analysis) with a significance level (alpha) of 0.05. The calculations assumed an unconditional (random X’s) model [23]. With taking into account any missing data we recruited a total of 1110 subjects to be included in the study. Inclusion criteria were: (a) age 65 years or older, (b) living independently, which was assessed by self-report. Participants were excluded if they had cognitive impairment that prevent them from providing informed consent, which was determined by a score below 24 on the Mini-Mental State Examination (MMSE). Participants with self-reported medical or neurological condition that prevented them from performing maximal HGS, or chronic conditions linked significantly to lower HGS were excluded. Written informed consent was obtained from all the participants. The study was approved by the ethical committee of the Prince Sattam Bin Abdulaziz University.

Sociodemographic data on age, gender, marital status, educational levels were collected. In addition, anthropometric measurements such as standing height, BMI, arm circumference, and upper arm length were collected. Upper arm circumference was measured in centimeters using measurement tape around the largest part of the upper arm. Similarly, the upper arm length was measured in centimeters using a measuring tape by measuring the distance from the acromion process in the shoulder joint to the olecranon process in the elbow joint.

### Hand grip strength (HGS)

The HGS was measured using a JAMAR PLUS+® digital hand dynamometer (Sam-mons Preston, Bolingbrook, IL, USA). Three trials were performed after one practice trial, for both hands. Dominant hand was determined by asking subjects if they were right or left handed. The average of the peak force of the three trials for the dominant hand was calculated by kilograms (kg). One-minute rest time was provided between trials. During testing, subjects were standing upright, with their feet hip-width apart and with elbow fully extended, and holding the hand dynamometer, with the testing wrist in neutral position and their index finger flexed at 90°. All data were collected by trained physical therapists.

### Statistical analysis

Data was analyzed using statistical software Stata version 15.1 (Stata Corp, College Station, TX). For continuous sociodemographic variables the mean and standard deviation were reported, and percentages were used for categorical variables. The normal distribution of variables was assessed by using Kolmogorov-Smirnov test. Descriptive data for the current sample was stratified by gender. Subjects’ sociodemographic and anthropometric characteristics were compared using U Mann-Whitney test for continuous variables and a chi-square test for categorical variables. A Spearman’s rank correlation was used to examine the relationship between sociodemographic and anthropometric data and HGS. A multiple linear regression analysis was used to assess predictors of the HGS. A *p*-value <.05 was considered significant.

## Results

A total of 1110 participants were included in this study. Sixty-two subjects were excluded due to missing data. Thus, data from 1048 participants (511 men, and 537 women) were used for statistical analysis. The sociodemographic and anthropometric characteristics were presented in Table 1. Level of education, height, arm length, weight, BMI, and arm circumference were statistically different between men and women (*p* < 0.05).

**Table 1:** Sociodemographic and anthropometric characteristics of the study sample

Variable	Men, *n* = 511	Women, *n* = 537	p
Age (years)	73.2 (5.3)	72.9 (5.2)	0.514
Education, n (%)			
No formal education	75 (14.7)	255 (47.4)	< 0.001
Elementary level	84 (16.4)	126 (23.4)	
High school	128 (25)	62 (11.5)	
Some college education	224 (43.8)	94 (17.5)	
Marital status, n (%)			
Married	446 (87.3)	453 (84.3)	0.176
Single	65 (12.7)	84 (15.6)	
Weight (Kg)	84.1 (18.1)	72.7 (15.9)	< 0.001
Height (cm)	172.4 (7.13)	157.6 (6.8)	< 0.001
BMI^a^ (Kg/m^2^)	28.2 (5.3)	29.2 (6.2)	0.004
Arm circumference (cm)	32.4 (4.2)	31.6 (4.7)	0.011
Arm length (cm)	38.9 (2.4)	35.6 (2.2)	< 0.001
Grip strength (kg)	34.37 (7.6)	21.03 (4.7)	< 0.001

Values are expressed as mean ± standard deviation; or numbers (%) when appropriate

^a^*BMI* Body Mass Index

Summary of the mean values with standard deviations and percentiles (5th – 95th) of the reference value and index of HGS from both genders are shown and divided by 5-years subsets in Table 2. The values of HGS in both genders were sequentially lower from the youngest age group (65–69 years) toward the older groups.

**Table 2:** Handgrip strength stratified by age groups, for both men and women

Age groups	Grip strength (kg)								
	n	Mean ± SD	P5 (95%CI)	P10 (95%CI)	P25 (95% CI)	P50 (95% CI)	P75 (95% CI)	P90 (95% CI)	P95 (95% CI)
Men									
65–69	192	36.9 **±** 8.3	21.1 (19, 23.2)	25.6 (22.4, 28.1)	31.8 (30.4, 33.3)	37.1 (36.2, 38.9)	42.3 (41, 43,5)	47.6 (46, 49.5)	50.1 (48.5,52.4)
70–74	121	35.7 **±** 7.4	25.2 (17.6,26.5)	26.5 (24.5, 28.8)	30.3 (29.2, 32)	36.2 (34, 37.2)	40.9 (39, 42.9)	45.1 (43.2, 48.7)	48.5 (45.4, 51.6)
75–80	198	30.5 **±** 7.1	19.2 (15.8,20.7)	21.4 (19.6, 23.2)	25.5 (24.4, 26.6)	31.0 (29.6, 31.9)	35.3 (33.9, 37.5)	39.6 (38.4, 41.4)	41.8 (40.3, 44.1)
Women									
65–69	206	23.2 **±** 4.7	15.3 (13.9, 16.7)	17.5 (15.9, 18.5)	20.2 (19.3, 21)	23.2 (22.3, 23.8)	25.9 (25, 27.1)	29.7 (28.7, 30.1)	30.4 (29.9, 32.7)
70–74	143	21.1 **±** 4.6	13.2 (10.2, 14.6)	14.8 (13.7, 16.1)	18.4 (16.7, 19.4)	21.1 (20.4, 21.8)	24.2 (23.2, 25.8)	27.2 (26.1, 28.5)	29 (27.2, 30.2)
75–80	188	18.8 **±** 4.9	9.9 (8.6, 11.3)	12.1 (10.3, 13.4)	15.5 (14.3, 16.7)	19.1 (18.1, 19.9)	22.1 (21.1, 23.7)	25.5 (24.6, 26.5)	26.6 (25.9, 28.5)

Table 3 shows the Spearman’s rank correlation coefficients between HGS and sociodemographic and anthropometric variables. The HGS negatively and significantly associated with the age in men (*r* = − 0.401, *p* < 0.001), and in women (*r* = − 0.386, *p* < 0.001). In addition, the HGS was positively and significantly associated with the weight, height, arm length and the arm circumference for both genders. Finally, the association between HGS and education and BMI was significant only in men.

**Table 3:** Spearman’s rank correlation coefficients between handgrip strength and other variables

Variable	Men		Women	
	r	p	r	p
Age (years)	−0.401	< 0.001	−0.386	< 0.001
Education	0.152	< 0.001	−0.021	0.629
Weight (Kg)	0.362	< 0.001	0.216	< 0.001
Height (cm)	0.384	< 0.001	0.339	< 0.001
BMI^*^ (Kg/m^2^)	0.232	< 0.001	0.072	0.094
Arm circumference (cm)	0.248	< 0.001	0.204	< 0.001
Arm length (cm)	0.418	< 0.001	0.154	0.001

Finally, a multiple linear regression analysis was performed. Age was the most important determinant of HGS in both gender, indicating that older participants tend to have lower HGS, where the progression in age by one year correspond to a reduction of HGS by 0.4 kg in men and 0.3 kg in women. In addition, moving from one educational level to a higher level correspond to an increase in HGS by 0.66 kg in men and a decrease by 0.42 kg in women. Also, participants with longer arms tend to have higher HGS, where an increase in arm length by one centimeter correspond to an increase of HGS by 0.87 kg in men (R^2^ = 0.35 for men, R^2^ = 0.26 for women). Finally, the analysis indicated non-significant association of the HGS with the participants’ weight, height, BMI, or the arm circumference for both genders (Table 4).

**Table 4:** Association between handgrip strength and other variables

Variable	Men			Women		
	Beta coefficient	95% CI	P	Beta coefficient	95% CI	P
Age	−0.40	− 0.52,-0.29	< 0.001	− 0.30	− 0.38, − 0.22	< 0.001
Education	0.66	0.10,1.21	0.021	− 0.42	− 0.75, − 0.08	0.013
Weight	0.11	− 0.35, 0.57	0.637	0.26	−0.06, 0.60	0.114
Height	0.26	−0.18, 0.72	0.252	0.01	−0.28, 0.32	0.903
BMI^a^	−0.68	−2.07, 0.71	0.334	−0.65	−1.47, 0.17	0.119
Arm circumference	−0.28	−0.65, 0.08	0.127	−0.05	− 0.31, 0.19	0.658
Arm length	0.87	0.60, 1.15	< 0.001	0.08	−0.08, 0.25	0.340

^a^*BMI* Body Mass Index

R^2^ = 0.35 for men

R^2^ = 0.26 for women

## Discussion

This study established the normal values for HGS in older adults living in Saudi Arabia, and explored the associated factors with HGS in this population. This study found that age was negatively associated with grip strength, and educational level was positively associated with grip strength in men but negatively in women. Men show positive association between arm length and HGS. This study is the first that established normal values of HGS for older adults in Saudi Arabia.

The results of this study were consistent with previous studies in different populations. The mean value in the current study for HGS in men was 34.37 kg, and it was consistent with studies in Turkey [24], Brazil [15], and China [16]. Although these studies reported similar findings with men, different measurements approaches were used, such as using maximum value of two measurement for both hands and different device [16]. The mean value of HGS in the current study for women was 21.03 kg, and it was consistent with previous reports in different countries [16–19, 24, 25]. Although all previous studies were performed in western countries that may have different associated factors such as race, weight and height, our results were consistent with some studies in terms of the mean value for HGS in men and women. Future work should investigate the underlying mechanisms of this association in Saudi population.

The current study found that age and educational level were associated with HGS in men and women. Our findings are consistent with some of previous studies [26, 27]. Prior evidence found that increased age was associated with decreased HGS [26–28]. As aging process affects multiple systems including musculoskeletal, nervous and vascular systems, these degenerative changes affect hand function and structure [29]. Past research found an association between aging and reduction in muscle mass as well as the ability to activate muscles [30]. Our study found that education was associated with HGS positively in men and negatively in women. This was partially consistent with a study from Singapore that found association between educational level and HGS in men older adults [31]. A recent meta-analysis reported that people with lower educational level had lower HGS [32]. Further research is needed to examine the underlying mechanisms of the association between level of education and HGS.

The present study found an increase in arm length that was associated with an increase in HGS in men. This was in partial agreement with a previous study that found this association [18]. However, in addition to men, this study found correlation between arm length and HGS in women. This could be attributed to different analytical approaches as this study used correlation while our study used multivariable regression analyses. Another factor is the height and arm length that could be different between countries and regions.

Few studies were conducted in the Middle East measuring HGS in adults. Although only one study included people in the Middle East aged 61 to 70 as subgroup [6], the average HGS for men (36) was different than the average of the current study (34.37). However, when HGS for men in this study (36) was compared with average HGS for men aged 65 to 69 in our study, the results were similar. The results of HGS for women in the middle East were 22 and these were partially consistent with our study results when compare HGS that was 23.2 for only women aged 65 to 69 years. These differences could be attributed to the age range differences and testing procedures as this study performed the test in sitting position while our study in standing position. Finally, our study used the average of 3 test trials for the dominant hand while the previous study used only results from one trial for the dominant hand. Further, using an average of three trials was found to be more reliable than using a single trial [33]. Overall, our study reported the normal values of HGS for older adults aged from 65 to 80 years while the other study in the middle east was limited to up to 70 years.

This study has some limitations. The lack of controlling for chronic comorbidities and hospitalizations in the last six months is one of the limitations needs to be acknowledged. These comorbidities could negatively affect HGS such as musculoskeletal diseases (e.g. hands arthritis). Therefore, the current findings should be interpreted with caution. Another limitation is that we didn’t used knee height as it has been shown to be an accurate surrogate measure of height in older adults, instead we used standing height. Although this study included a relatively large sample size, the generalizability is limited because the sample was limited to only older adults living in the Riyadh region and cannot be generalized to Saudi population. Occupation or previous occupation was not considered in this study and may affect the results. Future research is required to validate the current findings at a population-based level in Saudi Arabia.

## Conclusion

This study established the normal value for HGS and stratified by gender and age groups in older adults living in the Riyadh region in Saudi Arabia. This study found that age and educational level were associated with HGS in Saudi men and women, and arm length was associated with HGS in Saudi men.

## Data Availability

Data are available upon from the corresponding author on reasonable request.

## Abbreviations

HGS: Hand grip strength BMI Body mass index Kg Kilograms
